# A Systematic Review on the Role of Vitamin C in Tissue Healing

**DOI:** 10.3390/antiox11081605

**Published:** 2022-08-19

**Authors:** Nada Bechara, Victoria M. Flood, Jenny E. Gunton

**Affiliations:** 1 Centre for Diabetes, Obesity and Endocrinology Research (CDOER), The Westmead Institute for Medical Research, Lands of the Dharug Nation, The University of Sydney, Sydney, NSW 2145, Australia; 2 Department of Diabetes and Endocrinology, Blacktown-Mt Druitt Hospital, Sydney, NSW 2148, Australia; 3 Westmead Hospital, Sydney Medical School, Faculty of Medicine and Health, The University of Sydney, Sydney, NSW 2145, Australia; 4 University Centre for Rural Health, Faculty of Medicine and Health, University of Sydney, Lismore, NSW 2480, Australia; 5 School of Health Sciences, Faculty of Medicine and Health, University of Sydney, Sydney, NSW 2050, Australia

**Keywords:** vitamin C, ascorbic acid, healing, wound healing, tissue healing, supplementation

## Abstract

Vitamin C is an essential nutrient for humans and animals which are unable to synthesise it themselves. Vitamin C is important for tissue regeneration due to the role it plays in collagen formation, and its antioxidant properties. We reviewed the literature to evaluate potential associations between vitamin C supplementation and healing of an acute or chronic condition. Embase, Medline, PubMed, and the Cochrane Library were searched for studies published prior to April 2022. Studies were eligible if they reported at least one association between vitamin C supplementation and healing outcomes. Eighteen studies met the inclusion criteria and were included in this review. Overall, vitamin C supplementation improved healing outcomes in certain pathologies, predominantly pressure ulcers. However, many of the studies had small sample sizes, combined nutritional treatments, and did not test baseline vitamin C. Future studies should be of larger scale, exclusively using vitamin C to determine its role in tissue healing in other wounds. We recommend consideration of vitamin C supplementation for people with pressure ulcers.

## 1. Introduction

Vitamin C is a water-soluble substance found in the diet which is essential for life in humans [1]. Most animals can synthesise vitamin C from D-glucose, however humans and other primates, guinea pigs, fruit bats, some birds, some fish, and some insects, are unable to do so. These animals lack the key enzyme, gulonolactone oxidase, which is necessary for vitamin C biosynthesis [2]. Vitamin C is among the most unstable of all vitamins, with the cooking, handling and storage process decreasing the content in fruits and vegetables [3].

Tissue repair and regeneration within the body are influenced by vitamin C. A critical role of vitamin C is the synthesis of connective tissue, particularly collagen [1]. It also provides tensile strength to new collagen formed which would otherwise be unable to stretch without tearing [1]. In addition, vitamin C is an important antioxidant which can remove and neutralise oxidants in the body [4]. This is particularly important in the epidermis.

Along with its role in collagen synthesis, there is also evidence that vitamin C increases the proliferation of dermal fibroblasts, a function important for wound healing [4]. Vitamin C is consumed in these processes, so there is likely to be increased turnover at wound sites, suggesting that supplementation may be beneficial in healing. In addition, inflammation probably hastens the degradation and consumption of vitamin C; inflammation is normally present to at least a small degree in all wounds, with increasing inflammation in the setting of infection.

A deficient state of vitamin C is called scurvy. Scurvy can be fatal, with symptoms ranging from weakness, fatigue, malaise, red swollen gums, and bruising to infection, haemorrhage, bone fractures, and death [1]. In wound healing, delayed healing and impaired subcutaneous healing are associated with vitamin C deficiency [4].

In Australia, there is a surprising lack of data on vitamin C deficiency rates despite its importance for bodily functions. A study in 2018 found that 21.4% of 309 adults attending a surgical practice were deficient [5]. We recently reported a 50% deficiency rate in our high-risk foot service [6] and frequent deficiency in our diabetes clinic [7] and periodontal clinic [8]. Recommended dietary intakes (RDI) and estimated average requirements (EAR) of vitamin C are listen in Table 1.

This review will discuss the role that vitamin C plays in the healing of wounds, including surgical wounds, and the data around supplementation and healing.

## 2. Materials and Methods

The following databases were searched to identify relevant studies for inclusion:

(i) Cochrane Library (searched 26 April 2022)
(ii) Ovid Embase (searched 5 April 2022)
(iii) Ovid MEDLINE (searched 26 April 2022)
(iv) PubMed (searched 5 April 2022)

The combination of search terms used was as follows:

(vitamin C OR ascorbic acid) AND (wound healing).

The search was limited to “Humans” and “English” only. No limitations were placed on the date of publication.

One author (N.B.) screened the search results. The titles and abstracts were screened for all databases. The full manuscripts for papers that may have been relevant were read. The reference lists for these papers were also examined for potential studies to be included in this review, and one was found for potential inclusion; however, we were unable to obtain the full text.

**Figure 1:**
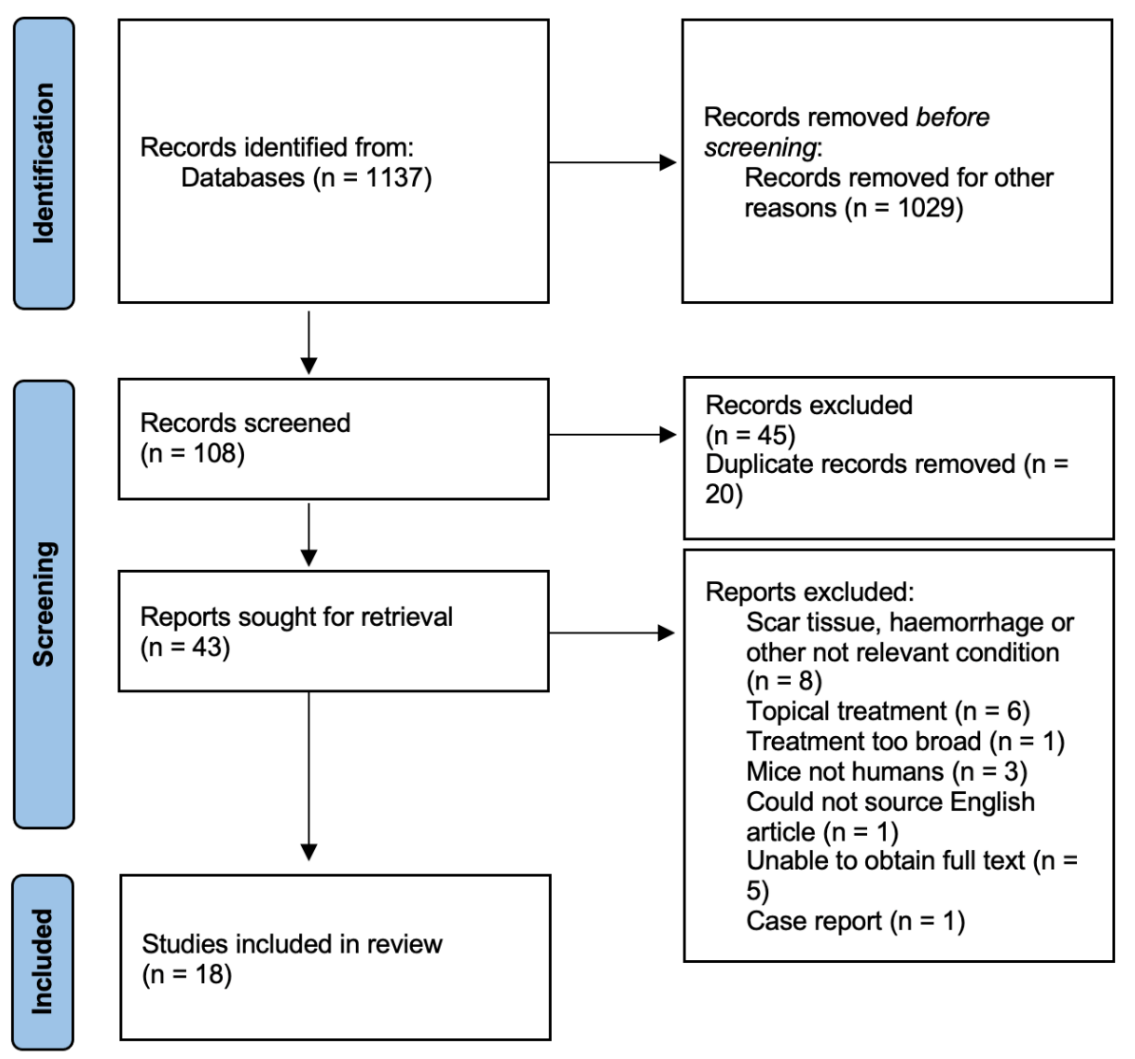
Identification of studies via databases.

The search process is outlined in Figure 1. 

### Inclusion and Exclusion Criteria

The studies to be reviewed had to meet the following criteria for inclusion: (a) to be randomised controlled trials, cohort studies or case-control studies, and (b) to include analysis of the relationship between treatment with vitamin C/ascorbic acid and any type of tissue healing in humans, including skin lesions, inflammation, and bone fractures. As mentioned above, papers not written in English were excluded.

Systematic review articles and case reports were not included. For the study to be included, there needed to be at least one outcome reported between vitamin C/ascorbic acid and the healing of an acute or chronic condition. Studies were excluded if treatment was administered topically, or if treatment targeted scar tissue. The identified studies used orally administered vitamin C.

## 3. Results

### 3.1. Study Selection

We initially identified 1137 studies from our database searches. Of these, 108 were screened based on their title and abstracts. Forty-five were then excluded and 20 were duplicates. Forty-three articles were selected for full-text reading, and, of these, 18 were relevant for inclusion. The criteria for exclusion are listed in Figure 1.

### 3.2. Description of Included Studies

Among the 18 included studies, there were six studies treating pressure ulcers, with a total of 234 patients [9,10,11,12,13,14]. Three studies involved a total of 233 people receiving treatment for dental extractions [15,16,17]. Two studies involved a total of 41 patients receiving treatment for foot ulceration [6,18]. In addition, there was one study that involved 128 patients receiving treatment for dental implants, and one with 10 patients receiving treatment for gingivitis [19,20]. One study involved 336 patients treated for acute fractures [21], one study considered the treatment of 32 children for burns [22], one involved 20 patients receiving treatment for unspecified trauma [23], one involved 21 patients receiving treatments for a hernia and the remaining study involved 40 patients undergoing a tattoo resection [24,25] ( Table 2 and Table 3).

Nine of the 18 included studies used additional components in their treatments, and the other nine used vitamin C alone. Studies with multiple components in their treatments are listed in Table 3.

### 3.3. Vitamin C Studies

#### 3.3.1. Pressure Ulcers

Six of the included studies treated pressure ulcers. Taylor et al. performed a prospective double-blind randomized controlled trial (RCT) with 20 patients receiving either 500 mg of ascorbic acid or an identical placebo twice daily for four weeks. The pressure ulcers were assessed weekly by an independent team using photographic measurements. After two weeks the vitamin C levels of all patients in the vitamin C group had significantly increased and were also greater than those in the placebo group [13]. The increased levels were also maintained throughout the length of the trial. The mean percentage reduction in ulcer size was 42.7% (*p* = 0.001) in the placebo group and significantly greater at 84% (*p* < 0.005) in the treatment group. The results were significantly better in the vitamin C group.

Ter Riet et al. performed a multi-site double-blind RCT of 89 subjects. Forty three received 500 mg of vitamin C and 45 received a placebo of 10 mg vitamin C [14]. The trial was run for 12 weeks with doses administered twice daily. The ulcer size and healing rates were calculated using a specialised computer program. Vitamin C increased in both groups by the end of the study, but without significant difference. Baseline levels were not specified. The mean healing rates were 0.21 cm^2^/week in the intervention group and 0.27 cm^2^ in the control group. There were no favourable outcomes for the vitamin C supplementation group.

#### 3.3.2. Dental Extractions, Implants and Gingivitis

Abrahmsohn et al. completed a double-blind RCT with 161 patients receiving treatment following a tooth extraction. The treatment group of 81 patients received 1500 mg vitamin C daily for three weeks [15]. Eighty patients in the placebo group received identical tablets. Patients taking vitamin C had a significantly shorter time to healing (*p* = 0.01).

Pisalsitsakul et al. performed a double-blind randomised controlled trial with 32 patients following a tooth extraction. The study consisted of three paired groups with a total of 128 extraction sites analysed [16]. The groups were placebo and administration of 600 mg vitamin C or 1500 mg vitamin C. All dosages were taken daily. The 600 mg group demonstrated a significant reduction in the extraction wound size compared with the placebo group (57.3% vs. 48.3%) at one week (*p* = 0.036). However, the 1500 mg group showed only a trend to decreased ulcer depth at 7 and 21 days (*p* < 0.07 for both compared to placebo).

Li et al. performed a randomised controlled trial considering the treatment of 128 patients who received a dental implant. Four implant techniques were evaluated, each divided into two subgroups—one sub-group received 300 mg vitamin C daily and the other sub-group served as a control [19]. Vitamin C increased wound healing at 14 days post-operatively in patients with chronic periodontitis and patients treated with a guided bone regeneration technique. However, vitamin C supplementation showed no benefits in patients treated with dental implants alone.

Woolfe et al. completed an RCT of 10 patients who were treated for gingival tissue. Patients received 250 mg of vitamin C, four times a day, or placebo, for six weeks [20]. There were no significant differences between the treatment and placebo groups. Unexpectedly, no significant differences were observed in serum vitamin C between the groups from the beginning to the end of the study period.

#### 3.3.3. Foot Ulcers

We conducted a double-blind pilot RCT of sixteen people with foot ulcers attending a foot ulcer clinic. Nine were randomised to receive a placebo and seven to receive 500 mg of slow-release vitamin C daily [6]. The pre-specified primary outcome of this study was the percentage of ulcers healing at eight weeks as measured by a specialised wound camera. Fifty percent of subjects had baseline vitamin C deficiency, with half of these showing undetectable levels. Ulcer healing at eight weeks was significantly higher in the vitamin C group compared to placebo (*p* = 0.041). At eight weeks, there were also no amputations in the vitamin C group compared to four in the control group.

#### 3.3.4. Fractures

Ekrol et al. performed a double-blind RCT of 336 patients who were treated for an acute fracture of the distal aspect of the radius. Of these patients, 186 had a displaced fracture and 150 had a non-displaced fracture [22]. Half of each cohort was randomised to receive 500 mg vitamin C, and the other half to receive the placebo, daily for fifty days after the fracture. At six weeks, patients in the treatment group with a non-displaced fracture showed significantly greater wrist flexion deficit (*p* = 0.008) and pinch strength deficit (*p* = 0.020). There was no significant difference in the time to fracture healing.

### 3.4. Studies with Combined Treatments

#### 3.4.1. Pressure Ulcers

Cereda et al. performed a multi-site randomised controlled trial with 28 patients receiving a nutritional supplement or placebo taken daily for 12 weeks. Patients who were fed orally were provided a total of 500 kcal, 34 g protein, 6 g arginine, 500 mg vitamin C, and 18 mg zinc [10]. Patients who were tube fed were provided 1000 mL of a high protein formula with 55 g protein, 8.5 g arginine, 380 mg vitamin C, and 20 mg zinc. The placebo condition for both groups was a standard hospital diet or a standard tube feeding formula. Ulcer healing was assessed using the Pressure Ulcer Scale for Healing (PUSH) tool. This considers the surface area, amount of exudate, and tissue at the base of the ulcer. There was significantly greater reduction in the ulcer area from week 8 and in PUSH scores at week 12. Overall, the patients treated with the nutrition formula showed a significantly higher mean reduction in pressure ulcer area (*p* < 0.005). Both groups showed a significant increase in dietary intake of vitamin C throughout the study (*p* < 0.001).

Desneves et al. performed an RCT with 16 patients treated for pressure ulcers. Patients were randomised to one of three groups: a standard hospital diet; a standard diet plus two high-protein energy supplements, or a standard diet plus two high protein energy supplements containing an additional 9 g arginine, 500 mg vitamin C and 30 mg zinc [11]. Pressure ulcer severity was measured using the PUSH tool over three weeks. Only patients receiving the additional C, zinc and arginine demonstrated a significant improvement in ulcer healing (*p* < 0.01). There were no significant changes in biochemical markers in any group; however, vitamin C levels showed a trend for improvement from baseline to week 3 in patients receiving the additional nutrients.

In another RCT, Van Anholt et al. considered the treatment of 43 patients for pressure ulcers over eight weeks. The treatment group received a 200 mL supplement, three times per day, each dose containing 250 kcal, 20 g protein, 3 g arginine, 250 mg vitamin C, 38 mg vitamin E, 238 mg vitamin A, 9 mg zinc, 64 mg selenium, 1.35 mg copper, and 200 mg folic acid [9]. The placebo condition was administration of a non-caloric placebo with similar appearance and taste to the treatment. Ulcer healing was significantly better in the nutritional supplement group (*p* < 0.016). PUSH scores in the supplement group were also significantly better than for the controls (*p* = 0.033). Serum vitamin C was significantly increased in the supplement group compared with the control group following the eight week treatment period (*p* = 0.015).

Frias Soriano et al. completed a prospective cohort study involving the treatment of 39 patients with pressure sores. Patients received an oral nutritional supplement daily for three weeks, comprising 250 kcal, 20 g protein, 3 g arginine, 250 mg vitamin C, 37.6 mg vitamin E, and 9 mg of zinc [12]. The condition and area of the ulcers were assessed weekly. After three weeks of supplementation, the median wound area reduced significantly (*p* < 0.001) from 23.6 cm^2^ to 19.2 cm^2^, representing a reduction of 29%. The amount of exudate in the infected ulcers (*p* = 0.012) and necrotic tissue (*p* = 0.001) also reduced significantly.

#### 3.4.2. Foot Ulcers

Yarahmadi et al. performed a randomised controlled trial on 25 patients with non-healing foot ulcers. Patients were treated with a platelet-rich plasma-fibrin glue (PRP-FG) dressing plus oral vitamin E and C or PRP-FG dressing plus placebo for eight weeks [18]. Eight weeks after treatment ulcer size was significantly reduced in both the intervention and control groups (*p* < 0.05). The reduction in wound size was significantly greater in the intervention group compared to the control group (*p* = 0.019). There was also a significant decrease in ESR and CRP levels noted in the intervention group compared to the control group (*p* < 0.05).

#### 3.4.3. Burns

Barbosa et al. performed a double-blind randomised controlled trial with 32 children receiving treatment for burns. Seventeen patients received an antioxidant mixture of vitamin C (1.5 times upper intake level), vitamin E (1.35 times upper intake level), and zinc (2.0 times recommended dietary allowance) for seven days starting on the second day of admittance into the hospital [22]. There was a significantly reduced time to wound closure in the treatment group compared to the placebo group (5.3 days vs. 7.5 days; *p* < 0.001). Vitamin C levels showed no significant differences in patients at completion of the study.

#### 3.4.4. Unspecified Trauma

Blass et al. completed a double-blind RCT with 20 trauma patients who had delayed wound healing, defined as persistent exudate 10 days after trauma or surgery. The groups received either an oral supplement providing 500 mg vitamin C, 166 mg vitamin E, 3.2 mg vitamin A, 100 mg selenium, 6.6 mg zinc, and 20 g glutamine, or placebo, for two weeks [23]. Micronutrient blood levels did not change, except for selenium, which increased in the treatment group (*p* = 0.009), and glutamine, which decreased in the placebo group (*p* = 0.047). Oxygen saturation decreased in the placebo group (*p* = 0.043) and wound closure occurred more rapidly in the treatment group when compared to controls (*p* = 0.01).

#### 3.4.5. Hernia Repair

Kjaer et al. performed an RCT with 21 men undergoing a hernia repair. The supplement group received 14 g arginine, 14 g glutamine, 1250 mg vitamin C, and 55 mg zinc daily, starting two weeks before surgery and ceasing two weeks after surgery [24]. Serum type 5 procollagen propeptide concentrations decreased (*p* < 0.05) post-operatively in the control but not the treatment group. Neither type 1 procollagen propeptide or type 3 procollagen propeptide serum concentrations differed between the two groups. In the wound discharge, the type 1 procollagen propeptide concentrations were 49% higher than those in the control group on day 2 (*p* = 0.10). Wound fluid concentrations of type 3 and type 5 procollagen propeptides showed no significant differences between groups. There were also no differences in time to wound healing.

#### 3.4.6. Tattoo Resection

Vaxman et al. completed a double-blind RCT in 49 patients undergoing a surgical resection of their tattoos. Eighteen patients received three-week supplementation of 1000 mg vitamin C combined with 0.2 g pantothenic acid [25]. In the treatment group, it was found that iron increased (*p* < 0.05) and manganese decreased (*p* < 0.05) in the skin. In the surgical scars on day 21, copper (*p* = 0.07) and manganese (*p* < 0.01) decreased, and magnesium (*p* < 0.05) increased. Serum vitamin C levels increased with supplementation after the surgery, whereas it decreased in the controls. All patients healed promptly and there were no differences in healing rates.

## 4. Discussion

Nutritional interventions have been shown to improve healing outcomes in certain pathologies. However, the role of vitamin C in specific wound types remains unclear. In this qualitative literature review, 18 studies were included which explored the effects of oral vitamin C administration on treatment outcomes.

For pressure ulcers, two studies assessed vitamin C alone and only one reported an improvement in ulcer outcomes [13]. However, the study which did not have significant results treated the placebo group with a low dose of vitamin C during the trial period [14]. The four studies which treated pressure ulcers combined with nutritional supplements all reported improved ulcer outcomes at the end of the trial period [9,10,11,12]. All four included protein, arginine, vitamin C and zinc. One of the studies also contained vitamin E, and the other, vitamin E, vitamin A, selenium, copper, and folic acid [9,12]. PUSH scores and ulcer area were significantly improved in all four studies. Two of the four studies reported on serum vitamin C levels and both reported an improvement at the end of the study period; however, only one was significant [9,11].

For dental ailments, all four studies treated patients with vitamin C alone, with three reporting favourable outcomes. One study reported a shorter time to healing following a tooth extraction when compared to the placebo group [15]. Pisalsitsakul et al. reported a reduction in extraction size between the 600 mg group; however, no difference between the 600 mg and 1500 mg vitamin C groups was observed, potentially because the vitamin C uptake may already have been saturated at 600 mg. In the study by Li et al., a significant effect on wound healing was only observed for chronic periodontitis with a guided bone regeneration technique. Vitamin C showed no benefit for dental implants alone, possibly due to the low dosage of 300 mg per day. In the study by Woolfe et al., there were no significant differences in either group, which may have been attributable to the very small sample size of 10 patients [20].

For foot ulcers, one study involved the use of vitamin C alone, whilst the other considered a combined treatment. Both studies reported favourable outcomes for wound healing [6,18]. One study reported that ulcer healing at eight weeks was significantly better in the vitamin C group compared to the placebo group (*p* = 0.041) [6]. At eight weeks, there were also no amputations in the vitamin C group compared to four in the control group. In a study by Yarahmadi et al., the ulcer size was significantly reduced in both the intervention and control groups (*p* < 0.05); however, the reduction was significantly greater in the treatment group (*p* = 0.019). There was also a significant decrease in the ESR and CRP levels in the treatment group compared to the control group (*p* < 0.05). However, both studies were single centre trials with small sample sizes.

Burns were assessed by only one study with a combined treatment of vitamin C with vitamin E and zinc [22]. Lipid peroxidation (*p* = 0.006) decreased, and vitamin E (*p* = 0.016) increased in the treatment group. The time to wound healing was lower in the treatment group compared to the control group (5.3 days vs. 7.5 days (*p* < 0.001)). It was hypothesised that this occurred as a result of the antioxidant protection provided by the vitamins and zinc against oxidative stress, facilitating a shorter time to wound healing. There was also a small increase noted in serum vitamin C levels in the supplementation group; however, the result was not significant.

Only one study treated fractures—specifically, patients with distal radius fractures [21]. In terms of functional outcomes at one year, there were no significant differences observed between the treatment or placebo groups in both displaced and non-displaced fractures. There were also no differences in time to fracture healing. It is important to note that serum vitamin C was not measured, and, therefore, that it is unknown whether those with a baseline vitamin C deficiency would benefit from supplementation following a fracture.

Blass et al. treated trauma patients with a combination of vitamin C, vitamin E, vitamin A, selenium, zinc, and glutamine [23]. Oxygen saturation decreased in the placebo group and wound closure occurred more rapidly in the treatment group. There were no significant differences in either group in serum vitamin C following the treatment period. This study confirmed that vitamin treatments may be beneficial in those with healing disorders.

The hernia repair study included a combined treatment with arginine, glutamine, and zinc [24]. The only significant findings were a decrease in type 5 procollagen propeptide concentrations post-operatively in the control but not in the treatment group, and an increase in type 1 procollagen propeptide concentrations on day 2 in the treatment group. These findings are of limited significance. The authors concluded that a larger trial is needed to confirm whether these early changes in the collagen profile translate into improved wound healing.

For the tattoo resection study, vitamin C was combined with pantothenic acid [25]. In the treatment group, fibroblast proliferation was increased, and mobilisation of trace elements was also observed, both of which are correlated with enhanced scar-tissue properties. These results suggest beneficial effects of vitamin C and pantothenic acid in healing. However, in this elective surgery group, all subjects healed well and there were no observed differences in healing.

Our review had several limitations, this included the heterogeneity of the reporting approach in the studies selected, many of which used small sample sizes, lacked reporting on baseline nutritional status, and lacked consistency in outcome reporting. Furthermore, the lack of studies in treating foot ulcers, fractures, and burns made it difficult to support or refute the use of vitamin C supplementation for these types of pathologies. The effects of the additional components in the treatment outcomes are also unknown and we are unsure to what extent these affected the results.

## 5. Conclusions

Overall, higher quality evidence is required to determine the efficacy of vitamin C supplementation when treating various pathologies. Many of the studies did not measure baseline vitamin C levels and, therefore, it is unknown whether those patients with a baseline deficiency would benefit from supplementation when compared to patients with normal baseline vitamin C levels.

Based on the available studies regarding pressure ulcers, there is sufficient evidence to support the use of supplementation by vitamin C during treatment; however, since this patient group suffers from a high rate of malnutrition, a more comprehensive nutritional supplement may offer further benefits.

The studies in dental care were of small size but suggest that patients should be assessed for nutrient intake, and that vitamin C supplementation should be recommended where fruit and vegetable intake is not sufficient.

Similarly, for foot ulcers, the reviewed studies were of small size, but, with the high rates of baseline deficiency, we suggest that vitamin C therapy should be considered.

Future studies should include larger scale randomised controlled trials with vitamin C alone in the treatment arm. Vitamin C supplementation should be recommended in people who are deficient.

## Figures and Tables

**Table 1:** Recommended Daily Requirements for Vitamin C in Australia [3].

Age (Years)	EAR (mg/Day)	RDI (mg/Day)
1–8	25	35
9–18	28	40
>19	30	45

**Table 2:** Vitamin C Studies.

Author, Year, Country	Study Type	No. Cases vs. Controls	Dosage, Duration	Baseline C Measured?	Wound Type	Study Outcomes
Abrahmsohn et al., 1993, USA	RCT	81 vs. 80	3 weeks, 500 mg t.i.d.	No, diet score	Dental extraction	Vit. C group healed faster (*p* = 0.01). 1.2% in vit. C group had dry socket vs. 10% of placebo. 12.3% in vit. C group healed slowly vs. 37.5% in placebo. No differences with gender or age.
Ekrol et al., 2014, Scotland	RCT	169 vs. 167	500 mg daily for 50 days	No, diet score	Fracture of the distal radius	No difference in time to fracture-healing. In non-displaced fracture subgroup, greater wrist flexion deficit (*p* = 0.008), pinch strength deficit (*p* = 0.02) and proportion with higher pain-score in vit. C group at 6 weeks but not at other times. At 52 weeks, higher rate of complications (*p* = 0.043) and greater pain (*p* = 0.045) in displaced fracture C group. No mention of correction for multiple comparisons. Measures would not retain significance if this needs to be added.
Gunton et al., 2021, Australia	RCT	7 vs. 9	500 mg daily for 8 weeks	Yes, 50%deficient	Foot ulcers	Healing at 8 weeks (primary endpoint) sig. better in vit C group (*p* = 0.041). Time to 50% reduction in wound volume faster. Healing without amputation in all in vit. C group vs. 5 of 9 controls.
Li et al., 2018, China	RCT	65 vs. 63	300 mg daily for 1 week postop	No	Dental implants	C group sig. higher healing rates than controls at day 7 (*p* < 0.05). No difference in pain between groups.
Pisalsitsakul et al., 2022, Thailand	RCT	10 vs. 12	10 days, 600 mg or 1500 mg t.i.d.	No. Meal record 7 days after extraction	Dental extraction	Pain scores sig. lower in 600 mg group vs. placebo (*p* < 0.05). Greater reduction at extraction site deficit in 600 mg/d vs. placebo (*p* < 0.05). Trend to reduced wound depth in 1500 mg group versus placebo (*p* < 0.07).
Taylor et al., 1974, England	RCT	10 vs. 10	500 mg twice daily for 4 weeks	Yes, leucocyte levels, mean in placebo 24 μg/10^8^ cells and 22 in tx group.	Pressure ulcers	Increased vitamin C in treatment group (65.5 vs. 25.8). Mean reduction of ulcer of 42.7% in placebo vs. 84% in C group (*p* < 0.005).
Ter Riet et al., 1995, The Netherlands	RCT	43 vs. 45	500 mg twice daily for 12 weeks	Yes, but deficiencies at baseline NR	Pressure ulcers	Mean increase 14.9 mg/litre in tx group, placebo group rose by 4.8 mg/litre, No significant difference in healing rates, 0.21 cm^2^ in C and 0.27 cm^2^/week in controls.
Woolfe et al., 1984, U.S.A	RCT	5 vs. 5	250 mg 4× day for 6 weeks	Yes, not different at baseline or 6 weeks	Gingivitis	No sig. correlations between Gingival Index. Did not find an increase in serum vit. C levels between tx and controls.
Yingcharoenthana et al., 2021, Thailand	Single-blind RCT	10 vs. 10 vs. 10	600 mg daily for 2 weeks	No	Dental extraction	Reduction in socket depth sig. higher in C group vs. controls (*p* = 0.028). No difference in radiographic density of new bone formation between groups.

RCT = randomised controlled trial, t.i.d. = three times a day, tx = treatment, no. = number, NR = not reported, sig. = significant, Vit. C = vitamin C.

**Table 3:** Studies with Combined Treatments.

Author, Year, Country	Study Type	No. Cases vs. Controls	Dosage, Duration	Additional Treatments	Baseline C Measured	Wound Type	Study Outcome
Barbosa et al., 2009, Brazil	RCT	17 vs. 15	1 week, 1.5× UL, t.i.d.	Vit E and Zn	Yes, no sig. difference between groups	Burns	No differences in CRP. No. of days to complete tissue repair sig. lower in active group (*p* < 0.001).
Blass et al., 2012, Germany	RCT	10 vs. 10	2 weeks, 2 sachets bd, 500 mg vit C	Vit E, A, Selenium, Zn, Glutamine	Yes, no sig. difference between groups	Trauma patients	CRP decreased in placebo group (*p* = 0.037). Wound closure faster in active group (29 vs. 58 days) (*p* = 0.01). No change in LoS.
Cereda et al., 2009, Italy	RCT	13 vs. 15	Oral 500 mg, tube fed 380 mg vit C daily	Protein, Arginine, Zn	No. Food diary, no differences at baseline.	Pressure ulcers	Greater reduction in wound surface area at 8-week follow-up in active group (57% vs. 33%) (*p* < 0.02). Sig. reduction in PUSH scores vs. controls (*p* < 0.05).
Desneves et al., 2005, Australia	RCT	6 vs. 5 vs. 5	3 weeks, 2× day 72 mg OR 500 mg vit C	Zn, Arginine	Yes, no differences	Pressure ulcers	Increase in vitamin C in group 3. PUSH score at week 3 sig. lower in group treated with vit. C (*p* < 0.05) with sig. improvements in PU surface area (*p* < 0.01).
Frias Soriano et al., 2004, Spain	Prospective	39	1–3× day for 3 weeks; 250 mg vit C	Protein, Vit E, Zn	No	Pressure ulcers	Reduced wound area (*p* < 0.001), exudate in infected ulcers (*p* = 0.012) and amount of necrotic tissue (*p* = 0.001).
Kjaer et al., 2020, Denmark	RCT	11 vs. 10	4 weeks, 1250 mg vit C daily	Arginine, Glutamine, Zn	No	Hernia repair	Serum procollagen propeptide concentrations decreased post-op in control group vs. active group (*p* < 0.05).
Van Anholt et al., 2010, The Netherlands	RCT	22 vs. 21	250 t.i.d. for 8 weeks; 250 mg vit C	Vit A, E, Zn, Cu protein, sel, arginine, folate	Yes, 23 mmol/L in tx group vs. 19.8 in control, 60.2 vs. 26.6 at end of study	Pressure ulcers	Decreased ulcer size vs. controls (*p* < 0.016). Fewer dressing changes required per week (*p* < 0.045). Serum vit C sig. increased (*p* = 0.015).
Vaxman et al., 1995, France	RCT	18 vs. 22	1000 mg daily for 3 weeks	Pantothenic acid	Yes, no differences reported	Tattoo resection	Serum vit. C increased in active group vs. decreased in controls. No change in healing process.
Yarahmadi et al., 2021, Iran	RCT	13 vs. 12	250 mg 2× day for 8 weeks	Vit E and PRP-FG dressing	No	Foot ulcers	Wound size reduced in both groups, and sig. greater in active group (*p =* 0.019). Decrease in CRP in active group (*p* < 0.05).

vit = vitamin, Zn = zinc, CRP = C-reactive protein, LoS = length of stay, PU = pressure ulcer, PUSH = pressure ulcer scale for healing, PRP-FG = platelet-rich plasma-fibrin glue, sig. = significant, tx = treatment.

## Abbreviations

EAR	Estimated average requirement
PRP-FG	Platelet-rich plasma-fibrin glue
PUSH	Pressure Ulcer Scale for Healing
RCT	Randomized controlled trial
RDI	Recommended dietary intake
